# Pneumococcal Pneumolysin Induces DNA Damage and Cell Cycle Arrest

**DOI:** 10.1038/srep22972

**Published:** 2016-03-30

**Authors:** Prashant Rai, Fang He, Jimmy Kwang, Bevin P. Engelward, Vincent T.K. Chow

**Affiliations:** 1 Infectious Diseases Group, Singapore-MIT Alliance for Research & Technology, Singapore 138602; 2 Department of Microbiology and Immunology, Yong Loo Lin School of Medicine, National University of Singapore, Singapore 117545; 3 Animal Health Biotechnology, Temasek Life Sciences Laboratory, National University of Singapore, Singapore 117604; 4 Department of Biological Engineering, Massachusetts Institute of Technology, Cambridge, MA 02139, USA.

## Abstract

*Streptococcus pneumoniae* produces pneumolysin toxin as a key virulence factor against host cells. Pneumolysin is a cholesterol-dependent cytolysin (CDC) toxin that forms lytic pores in host membranes and mediates pneumococcal disease pathogenesis by modulating inflammatory responses. Here, we show that pneumolysin, which is released during bacterial lysis, induces DNA double strand breaks (DSBs), as indicated by ataxia telangiectasia mutated (ATM)-mediated H2AX phosphorylation (γH2AX). Pneumolysin-induced γH2AX foci recruit mediator of DNA damage checkpoint 1 (MDC1) and p53 binding protein 1 (53BP1), to sites of DSBs. Importantly, results show that toxin-induced DNA damage precedes cell cycle arrest and causes apoptosis when DNA-dependent protein kinase (DNA-PK)-mediated non-homologous end joining is inhibited. Further, we observe that cells that were undergoing DNA replication harbored DSBs in greater frequency during pneumolysin treatment. This observation raises the possibility that DSBs might be arising as a result of replication fork breakdown. Additionally, neutralizing the oligomerization domain of pneumolysin with monoclonal antibody suppresses DNA damage and also cell cycle arrest, indicating that pneumolysin oligomerization is important for causing DNA damage. Taken together, this study reveals a previously unidentified ability of pneumolysin to induce cytotoxicity via DNA damage, with implications in the pathophysiology of *S. pneumoniae* infection.

Severe pneumonia caused by *Streptococcus pneumoniae* results in significant mortality due to various complications, including pulmonary edema secondary to alveolar-capillary barrier destruction^1^ and cardiovascular failure^1^,^2^,^3^. Intriguingly, complications can persist even after antibiotic intervention that eliminates the pneumococci^1^,^3^. These observations call attention to the potential for molecular components of pneumococci to induce cytotoxicity, rather than the live organism. As such, it is important to understand the host responses during bactericidal antibiotic treatment, since responses toward pneumococcal proteins that remain in circulation may influence disease severity and progression. Pneumolysin, a toxin produced by *S. pneumoniae*, is known to be released during enzyme-mediated autolysis^4^, competence-induced lysis^5^ and antibiotic-mediated lysis of pneumococci^6^. Importantly, pneumolysin has been shown to be crucial for pneumococcal virulence and invasion *in vivo*^7^,^8^. While pneumolysin-induced cytotoxicity is well-documented^9^,^10^,^11^, the molecular processes underlying its toxicity are not yet fully understood.

Pneumolysin belongs to a cholesterol-dependent cytolysin (CDC) family of toxins, which are produced by Gram-positive bacteria^1^. CDCs primarily mediate host cell death by binding to membrane cholesterol and then oligomerizing to form macromolecular pores that perforate the host membrane to mediate cell lysis^12^,^13^. In animal models of infection, the lytic activity of pneumolysin is associated with disruption of alveolar-capillary barrier, which exacerbates disease severity^14^,^15^. Indeed, purified pneumolysin can directly cause vascular leakage and edema^16^. Pneumolysin present in the systemic circulation has also been shown to cause myocardial injury during pneumococcal infection^17^. Moreover, pneumolysin triggers activation of pro-inflammatory immune cells, leading to release of reactive oxygen and nitrogen species that damage host tissues^18^,^19^. Taken together, these studies implicate pneumolysin as a key protein in the mediation of disease severity.

In addition to the ability to induce membrane-mediated toxicity and to activate immune responses, there is also accumulating evidence that CDC toxins play additional roles in modulating cell signaling and function^13^. Specifically, recent studies have called attention to infection-induced DNA damage as a possible pathogenicity factor^20^,^21^. Of particular interest is the impact of pneumolysin on formation of DNA double strand breaks (DSBs). DSBs are considered to be one of the most toxic forms of DNA damage as they can cause chromosomal rearrangements and even cell death if they are not properly repaired^22^. The process of DSBs repair is initiated by ATM kinase-mediated phosphorylation of histone variant H2AX to form γH2AX^23^. Foci of γH2AX at DSBs serve to recruit repair proteins such as 53BP1^24^ that facilitates repair of DSBs via non-homologous end joining (NHEJ) pathway^25^. During G1, NHEJ pathway^26^ is initiated when the heterodimer complex of Ku70-Ku80 binds to broken DNA ends. The Ku complex recruits catalytic DNA-PKc to form complete holoenzyme DNA-PK, which facilitates the processing of damaged termini by Artemis and ligase IV^27^. To prevent the deleterious effects of DSBs, in addition to facilitating repair, ATM can activate cell cycle arrest mainly via checkpoint kinase 2 (Chk2)^28^, allowing time for the repair of DSBs. Despite the presence of repair pathways and cell cycle arrest, under severe conditions, DNA damage can persist and drive the cellular response towards apoptosis^29^, thus eliminating cells that would otherwise suffer from genomic instability. Hence, the extent of DNA damage and the resultant cellular responses determine cell fate under stress.

Given that the pneumococcal toxin pneumolysin is associated with apoptosis^9^ and that DNA damage can trigger apoptosis^22^, here we investigated the possibility that pneumolysin causes DNA damage and its downstream sequelae. We found that even in the absence of live bacterial cells, pneumolysin is able to induce DSBs in alveolar epithelial cells. In addition, we also show that pneumolysin-induced DNA damage is associated with cell cycle arrest and that pneumolysin binding and oligomerization in the membrane plays significant role in its genotoxicity. Finally, results from these studies show that pneumolysin-induced DSBs give rise to repair foci and that inhibition of DSB repair exacerbates pneumolysin-induced toxicity, pointing to DNA repair as a potentially important susceptibility factor. Taken together, the results of these studies reveal that pneumolysin is genotoxic, thus providing a molecular mechanism that potentially contributes to pneumolysin’s pathogenicity.

## Results

### Pneumolysin induces DNA damage and cell lysis

**Figure 1:**
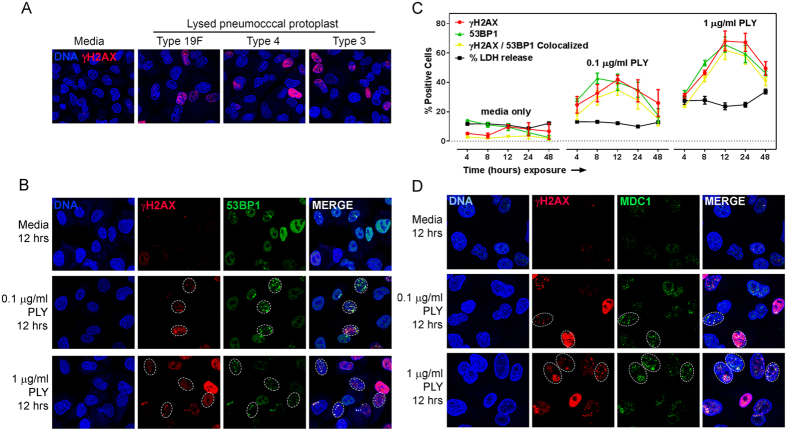
Pneumolysin induces cell DNA damage and cell lysis. (**A**) Lysate of pneumococcal protoplast induces DNA damage in alveolar epithelial cells. *S. pneumoniae s*erotypes 19F, 3 and 4, grown in bacteria media, are re-suspended in cell wall digestion buffer to isolate the protoplasts, which are then physically lysed by Bead beater in F12-K culture media. The pneumococcal lysates are then incubated with alveolar epithelial cells for 7 h and analyzed for γH2AX. Representative images of three independent experiments showing γH2AX (red) and nuclei (blue). (**B**,**C**) Pneumolysin induces double strand breaks (DSBs) in alveolar epithelial cells. (**B**) Recombinant pneumolysin was incubated with alveolar epithelial cells at 0.1 μg/ml and 1 μg/ml concentration for 12 h and analyzed for γH2AX and 53BP1. Representative images showing DSBs in alveolar epithelial cells with nuclei (blue), γH2AX (red), 53BP1 (green) and colocalized foci (yellow). (**C**) Alveolar epithelial cells exposed to pneumolysin were analyzed for γH2AX and 53BP1 at 4, 8, 12, 24 and 48 h of incubation. γH2AX and 53BP1 positive cells (≥5 foci per nucleus) were quantified and expressed as percentage positive cells. Cytotoxicity was quantified by using LDH assay and expressed as percent of total LDH release during lysis by 1% Triton X-100. (**D**) Representative images showing DSBs in alveolar epithelial cells with nuclei (blue), γH2AX (red), MDC1 (green) and colocalized foci (yellow). ‘Media’ indicates negative control without any pneumolysin. Results show mean ± SEM for three independent experiments.

Pneumolysin toxin is mostly found in the cytoplasm of *S. pneumoniae* and it cannot be actively secreted as it lacks a secretory signal^30^. Therefore, the biological relevance of pneumolysin is specific to lysed bacterial cells. To explore the ability of pneumolysin to cause DNA damage, we therefore first investigated whether pneumococcal lysate can induce host DNA damage. We exposed alveolar epithelial cells to lysate of pneumococcal protoplasts of three different serotypes, namely −19 F, 3 and 4 (Fig. 1A). The frequency of γH2AX foci, which form at sites of DSBs, was measured after exposure. We found that lysates from all three serotypes induced a significant increase in the frequency of γH2AX positive cells (≥5 foci per nucleus). These data raise the possibility that pneumolysin, which is released after lytic death of bacteria, can induce DNA repair foci.

To further probe the potential for pneumolysin to induce DNA damage, we created recombinant pneumolysin. Alveolar epithelial cells were exposed to recombinant pneumolysin (Fig. 1B) and analyzed to determine the levels of γH2AX foci and 53BP1 foci, a downstream DNA repair protein that frequently colocalizes with γH2AX^24^ and helps to direct the DSB repair toward non-homologous end joining^25^. We observed that pneumolysin induces discrete foci of γH2AX and 53BP1 in epithelial cells, in a concentration dependent manner. Visual inspection suggests that at 1 μg/ml, pneumolysin induced a greater number of DNA damaged cells than at 0.1 μg/ml (Fig. 1B). In addition, we observed that most γH2AX foci induced by pneumolysin colocalized with 53BP1, which is consistent with the formation of DSBs. We further investigated kinetics of the occurrence of DSBs in alveolar epithelial cells for up to 48 h of pneumolysin treatment (Fig. 1C). We found that recombinant pneumolysin was able to induce DSBs in significant percentage of cells as early as 4 h, with maximum DNA damage around 12 h. We also observed that at 12 h post-incubation, the higher concentration of pneumolysin (1 μg/ml) was able to induce DSBs in over 60% of host cells compared to ~40% induced by 0.1 μg/ml pneumolysin (Fig. 1C). Furthermore, the frequency of alveolar epithelial cells harboring DSBs decreased after ~12 h. Given that DSBs are able to activate repair processes as an integral part of DNA damage responses, the decline in the percentage of cells harboring DNA damage is consistent with DSBs repair. To further reveal the proteins that are present in the repair foci, we analyzed the pneumolysin-induced γH2AX foci for the presence of another DNA repair protein, namely MDC1. MDC1 binds to γH2AX^31^, creating a platform for recruitment of phospho-ATM, which leads to phosphorylation of various repair proteins, including 53BP1^31^. We observed that pneumolysin-induced γH2AX foci indeed colocalize with MDC1 protein, indicating activation of DNA damage responses (Fig. 1D). Consistent with accumulation of damage response proteins at pneumolysin-induced repair foci, we observed that 53BP1, which colocalized with γH2AX foci, is also phosphorylated (Supplementary Fig. 1). These data provide strong evidence that pneumolysin-induced γH2AX foci are indeed associated with DSBs.

There are many reports showing that pneumolysin disrupts the host cell membrane^9^,^11^. Hence, we also analyzed release of cytoplasmic lactate dehydrogenase (LDH) as an indicator of pneumolysin-induced membrane pores (Fig. 1C). We found that pneumolysin causes significant LDH release from alveolar epithelial cells at 1 μg/ml concentration but did not cause any detectable cell lysis at 0.1 μg/ml. Given that pneumolysin was genotoxic at 0.1 μg/ml, this result suggests that pneumolysin is able to induce DSBs without significantly disrupting the host cell membrane. Overall, these data suggest that pneumolysin released during pneumococcal lysis is able to induce discrete γH2AX foci as well as potential repair responses, even in the absence of significant cell lysis.

### Pneumolysin induces ATM and activates DNA-PK mediated repair

**Figure 2:**
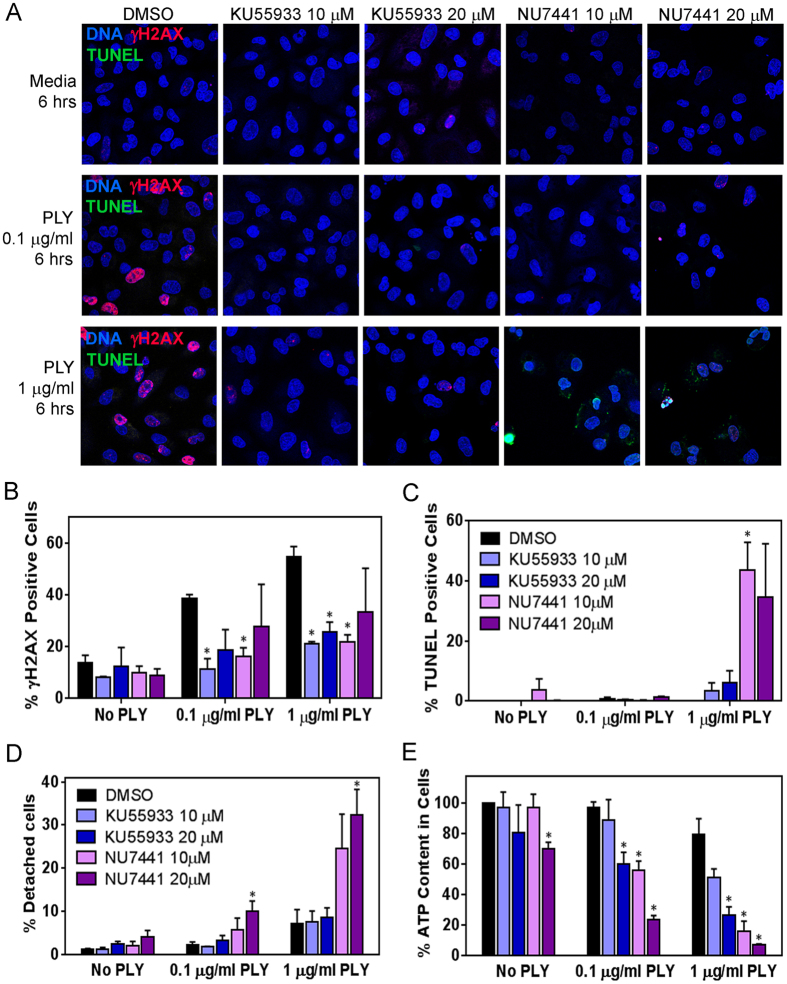
Pneumolysin-induced DNA damage response is mediated by ATM and activates DNA-PK-mediated repair. (**A**) Alveolar epithelial cells were incubated with pneumolysin at 0.1 μg/ml and 1 μg/ml concentration for 6 h, in the presence of 10 μM and 20 μM of ATM inhibitor (KU55933) or DNA-PK inhibitor (NU7441) in DMSO. Representative images showing inhibition of γH2AX formation by KU55933 and NU7441 as well as apoptosis induction by NU7441, with nuclei (blue), γH2AX (red) and TUNEL (green). Images are representative from four independent experiments. (**B**) γH2AX positive (≥5 foci per nucleus) and (**C**) TUNEL positive cells were quantified and expressed as percentage positive cells. (**D**) During pneumolysin treatment with KU55933 or NU7441, the numbers of detached cells in the culture medium were determined using hemocytometer and expressed as percentage of cells initially seeded (1 × 10^5^). (**E**) The overall ATP content in each well was determined and expressed as percentage of total ATP in the negative control (DMSO-treated, No PLY). Results show mean ± SEM for three independent experiments. *p < 0.05, unpaired Student’s *t*-test between DMSO and inhibitor treatment conditions.

To further reveal DNA damage responses during pneumolysin-induced DSBs, we studied phosphatidylinositol 3-kinase related kinases (PI3KK), which play important roles in response to DNA damage^27^. Specifically, DSBs activate response pathways that are regulated by PI3KK proteins, including ATM kinase, which phosphorylates H2AX^23^. To learn whether pneumolysin-induced DSBs activate the canonical ATM kinase pathway, we treated alveolar epithelial cells with an ATM kinase inhibitor (KU55933) at the same time as being exposed to pneumolysin (Fig. 2A). We found that inhibition of ATM kinase significantly reduces formation of γH2AX (Fig. 2B). We also observed that there were neither significant levels of apoptosis in the adherent epithelial cells (indicated by TUNEL staining) (Fig. 2C) nor detached cells in culture supernatant (Fig. 2D). However, there was a significant reduction in total cellular ATP content when epithelial cells were exposed to pneumolysin in presence of 20 μM KU55933 (Fig. 2E), indicating a decline in metabolic activity when the ATM pathway is inhibited. In addition, we also observed that the most genotoxic concentration of pneumolysin (1 μg/ml) led to a greater reduction in cellular ATP (Fig. 2E). These observations are consistent with pneumolysin-induced DNA damage activating the ATM response pathway, inhibition of which results in decreased γH2AX foci and metabolic activity.

DNA-PKcs is another member of the PI3KK family that plays an important role in the DNA damage response^32^. Specifically, DNA-PKcs interacts with Ku70 and Ku80 to form the DNA-PK holoenzyme, which is essential for repair of DSBs via the NHEJ pathway^27^. We challenged cells that were exposed to pneumolysin with exposure to the DNA-PKcs inhibitor NU7441 (Fig. 2A). We observed a significant reduction in pneumolysin-induced γH2AX formation, which is consistent with previous studies showing that a significant proportion of γH2AX results from phosphorylation by DNA-PKcs^32^ (Fig. 2B). Additionally, exposure to NU7441 resulted in increased apoptosis of alveolar epithelial cells (TUNEL positive) during exposure to high pneumolysin concentration (Fig. 2C). Furthermore, inhibition of DNA-PK during pneumolysin exposure resulted in a significant decrease in ATP levels in alveolar cells (Fig. 2E) and a parallel increase in number of detached cells in the culture supernatant (Fig. 2D). The observation of apoptotic cells and presence of detached cells in culture during NU7441 treatment suggests that the decrease in ATP levels is due to loss of viable cells. Overall, DNA-PK inhibition during pneumolysin-induced DNA damage exerted greater detrimental effect on cell survival than ATM inhibition. This may be attributed to the indispensable role of DNA-PK in NHEJ repair pathway, such that its inhibition impairs DNA repair and leads to cell death. Taken together, these findings indicate that pneumolysin-induced DSBs activate the ATM pathway. Finally, suppression of DNA DSB repair via inhibition of DNA-PKcs results in increased pneumolysin-induced cell death, pointing to the potential importance of pneumolysin-induced DSBs in cell fate.

### Pneumolysin induces cell cycle arrest

**Figure 3:**
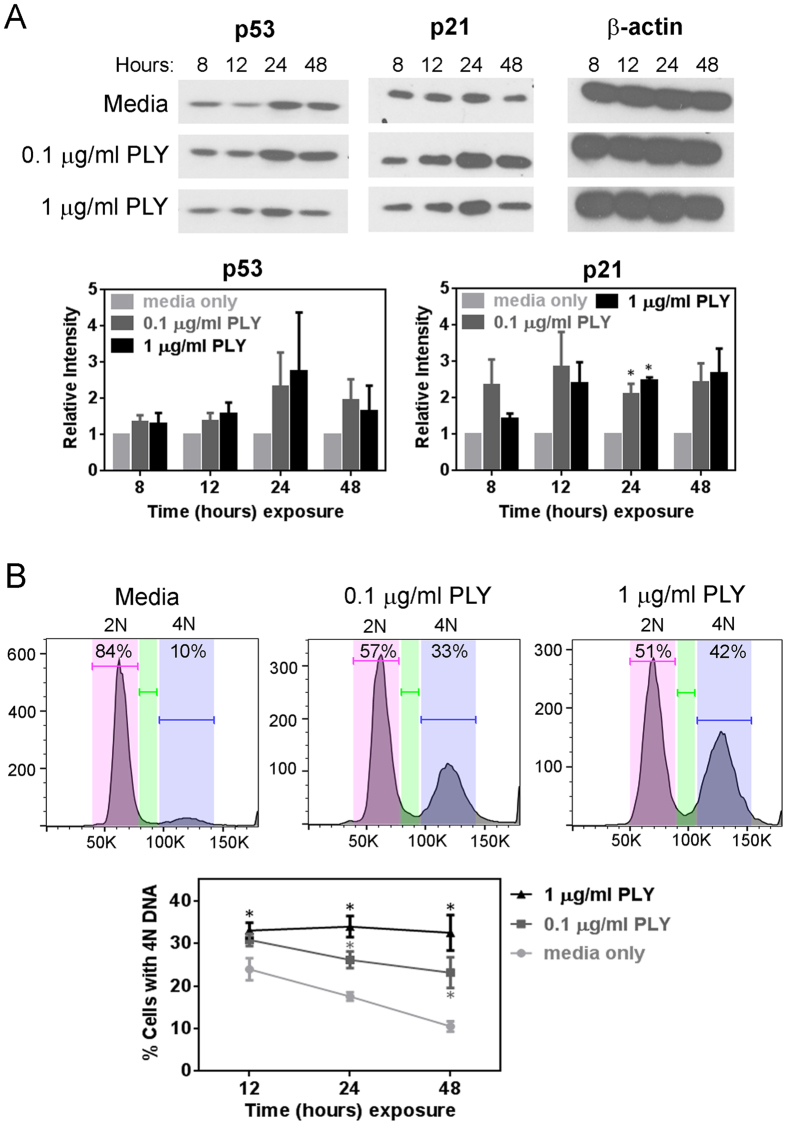
Pneumolysin-induced DNA damage is associated with cell cycle arrest. (**A**) Alveolar epithelial cells exposed to pneumolysin were lysed at different time-points during incubation and the lysate was probed for p53 and p21 by western blot. β-actin served as loading control. Each blot is representative of three independent experiments. Protein band intensity analysis was done on all three experiments and is expressed as fold change relative to ‘media only’ control after normalizing with β-actin. (**B**) Cells exposed to pneumolysin were also analyzed for cell cycle using propidium iodide. Representative histograms of three independent experiments showing alveolar epithelial cell arrest at 4N DNA content after treatment with pneumolysin for 48 h. Percentage cell cycle arrest at 12, 24 and 48 h was quantified. Results show mean ± SEM for three independent experiments. *p < 0.05, unpaired Student’s *t*-test.

In addition to the ability of pneumolysin-induced DNA damage to cause cell death during inhibition of DNA repair, we were also interested in the possibility that pneumolysin-induced DNA damage can affect cell cycle progression. DNA damage-induced p53 activation following exposure to relatively low levels of damage can lead to cell cycle arrest, allowing time for repair, whereas relatively high levels of damage can lead to p53 mediated apoptosis^33^. Given the pivotal role of p53 expression in cell fate^34^,^35^, first we examined the level of p53 protein during pneumolysin treatment. Figure 3A shows that there appears to be an increase in p53, however the result was not statistically significant (in terms of band intensity), Although we did not observe a significant increase in the levels of p53, it remained possible that downstream targets of p53 might still be activated. One of the important proteins that regulates cell cycle progression is p21, which inhibits cell cycle promoting cyclin-dependent kinases (CDK1 and CDK2^36^,^37^) to sustain cell arrest signals during DNA damage^38^. We found significantly increased levels of p21 at 24 h of pneumolysin treatment, indicating potential induction of cell cycle arrest (Fig. 3A). Indeed, when the cell cycle status of pneumolysin-treated cells was determined by analyzing their DNA content, we observed that a significant percentage of cells were arrested at 4N DNA content of late S-phase and G2/M transition phase cells at 24 and 48 hours (Fig. 3B). Similar to the pneumolysin-induced DNA damage, the extent of pneumolysin-induced cell arrest was also dependent on its concentration, with higher genotoxic concentration inducing greater percentage of arrested cells. Overall, these data suggest that pneumolysin-induced DSBs are associated with cell cycle arrest.

### Pneumolysin-induced DNA damage is associated with DNA replication

**Figure 4:**
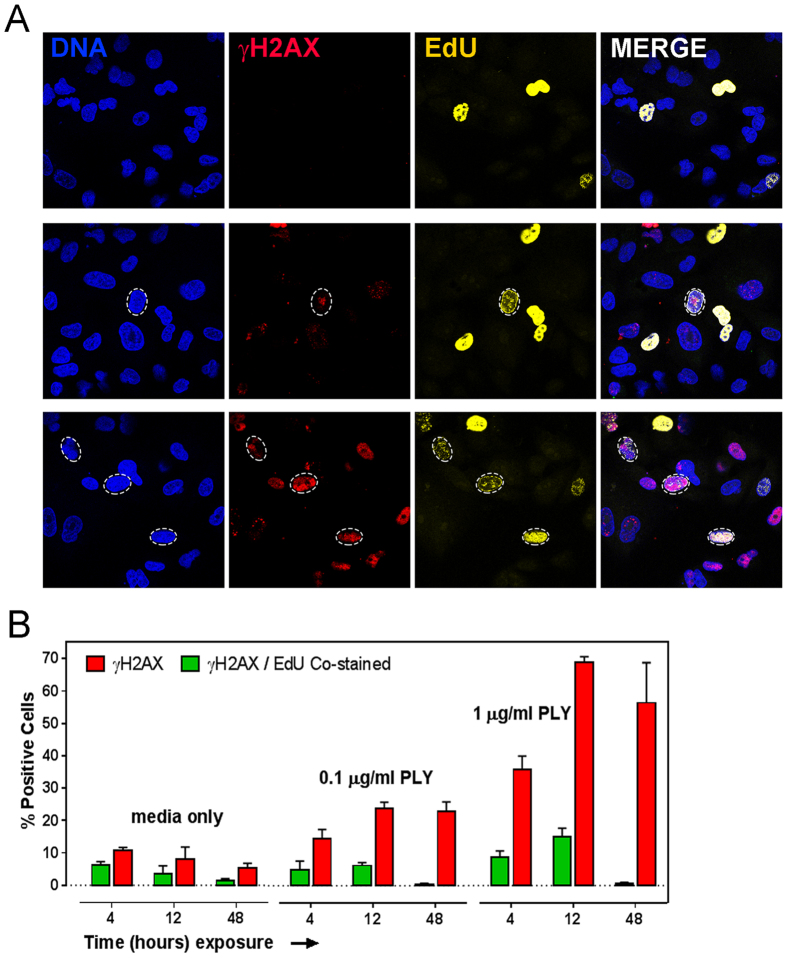
Association between pneumolysin-induced γH2AX and DNA replication. (**A**) Alveolar cells incubated with pneumolysin for 4, 12 and 48 h were labeled with EdU for 30 min at the end of each time point. The cells were then fixed and probed for γH2AX and EdU. Representative images showing nuclei (blue), γH2AX (red) and EdU (yellow). Images are representative from three independent experiments. (**B**) γH2AX, EdU positive (≥5 foci per nucleus) and dual-positive cells were quantified and expressed as percentage positive cells. ‘Media only’ indicates negative control without pneumolysin. Results show mean ± SEM for three independent experiments.

During DNA replication, replication forks can break down when they encounter a single strand break (SSB)^39^, which can cause the formation of a double strand end that can lead to H2AX phosphorylation. In addition, replication stress due to stalling or slowing of replication fork is also known to cause H2AX phosphorylation^40^. To learn whether pneumolysin-induced DNA damage is associated with DNA replication, pneumolysin exposed cells were pulsed with EdU for 30 minutes at intervals of 4, 12 and 48 h of exposure and fixed immediately to analyze for γH2AX and EdU (Fig. 4). Here, the population of γH2AX and EdU positive cells depicts actively replicating cells with DNA damage. At 12 h, we observed that ~26% of total γH2AX-positive cells, induced by 0.1 μg/ml pneumolysin, were EdU-positive (Fig. 4). Similarly, ~22% of γH2AX-positive cells were also EdU positive during 1 μg/ml pneumolysin treatment. These results indicate that during pneumolysin induced genotoxicity, a sub-population of DNA damaged cells are also undergoing DNA replication, raising the possibility that pneumolysin-induced DNA damage could be associated with replication stress or fork breakdown. At 48 h, we did not observe any significant number of cells that were positive for both replication and DNA damage (Fig. 4), possibly due to reduced replication as a result of cell arrest at 48 h (Fig. 3B). Taken together, these results suggest that pneumolysin-induced DNA damage is partly associated with DNA replication.

### Neutralizing pneumolysin prevents DNA damage and cell arrest

**Figure 5:**
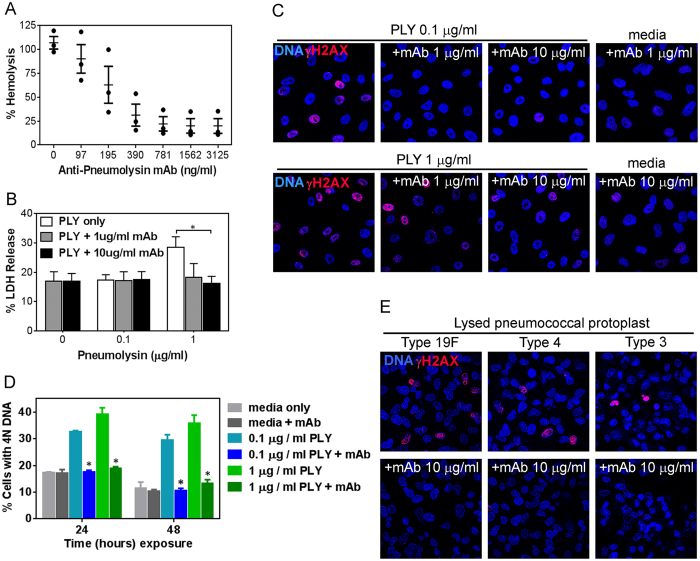
Neutralizing oligomerization domain of pneumolysin prevents its genotoxicity. (**A**) Hemolysis assay was performed to test the ability of anti-pneumolysin monoclonal antibody (mAb) to neutralize the lytic activity of 25 ng/ml pneumolysin against RBCs. Extent of hemoglobulin released during pneumolysin-mediated RBC lysis is expressed as percentage of total hemolysis during RBC treatment by lysis buffer. (**B**) Alveolar epithelial cells were exposed to pneumolysin for 12 h in the presence of anti-pneumolysin mAb. LDH assay was performed on the culture supernatant, and expressed as percentage of total LDH released during lysis by 1% Triton X-100. Similarly, alveolar epithelial cells exposed to pneumolysin, in the presence of anti-pneumolysin mAb, were analyzed for (**C**) γH2AX at 12 h and (**D**) cell cycle arrest at 24 h and 48 h. (**E**) Alveolar epithelial cells were exposed to pneumococcal lysate for 7 h in the presence of anti-pneumolysin mAb. Representative images from three independent experiments showing prevention of DNA damage by mAb, with nuclei (blue) and γH2AX (red). Results show mean ± SEM for three independent experiments. *p < 0.05, unpaired Student’s *t*-test.

Pneumolysin is known to make pores in cell membrane by oligomerization of 35–47 monomers that assemble into a pre-pore structure which undergoes conformational changes to form a complete 26 nm ring-shaped pore, characteristic of CDC family^12^. Given that oligomerization of pneumolysin is the initial process in eliciting cellular responses, we sought to understand the role of oligomerization in pneumolysin-induced DSBs. To learn about the impact of oligomerization, we generated a monoclonal antibody (mAb) against the oligomerizing domain of recombinant pneumolysin and we showed that it neutralizes the hemolytic ability of toxin by preventing oligomerization and subsequent pore formation in erythrocytes (Fig. 5A). We then tested the ability of the mAb to neutralize cytotoxicity of pneumolysin in alveolar epithelial cells and found that the mAb was able to reduce pneumolysin-induced LDH release from alveolar epithelial cells (Fig. 5B). We next asked about the impact of oligomerization on DNA damage. Remarkably, alveolar epithelial cells exposed to pneumolysin in the presence of mAb showed a significant reduction in the frequency of cells with DSBs, as indicated by γH2AX staining (Fig. 5C). In addition, pre-treatment of pneumolysin with cholesterol, which binds to pneumolysin and competitively inhibits its attachment to membrane cholesterol, also diminished DNA damage caused by pneumolysin (Supplementary Fig. 2). These observations show that neutralizing pneumolysin by either preventing its oligomerization in host membrane or preventing its initial binding to membrane cholesterol, attenuates its genotoxicity. Thus, these studies suggest that membrane binding and oligomerization are critical for pneumolysin-induced host cell DNA damage.

Having shown that neutralization of pneumolysin inhibits pneumolysin induced cytotoxicity and DNA damage, we next asked about the importance of oligomerization in cell cycle arrest. We found that neutralizing pneumolysin oligomerization not only reduced DNA damage (Fig. 5C), but it also prevented pneumolysin-induced cell cycle arrest (Fig. 5D). This observation is consistent with a model wherein pneumolysin-induced DSBs lead to cell cycle arrest. Further, to extend from recombinant pneumolysin to a more physiologically relevant condition, we studied genotoxicity associated with pneumococcal lysate. Alveolar epithelial cells were exposed to pneumococcal lysate, along with the mAb. Consistent with the results using recombinant pneumolysin, the presence of anti-pneumolysin neutralizing mAb significantly reduced the frequency of γH2AX positive cells (Fig. 5E), demonstrating that pneumolysin plays a critical role in mediating DNA damage when pneumococci undergo lytic death. Together, these data show that pneumolysin released during bacterial lysis is an important genotoxic factor in pneumococcal lysate, and that pneumolysin oligomerization and binding underpin its genotoxicity.

## Discussion

*S. pneumoniae* infection underlies 30–50% of pneumonia cases, amounting to 1–2 million deaths per year^41^. While antibiotics have been a mainstay for treating *S. pneumoniae* infection, antibiotic resistance is a growing problem, calling attention to the need for alternative approaches for disease mitigation. Here, to explore strategies for treating pneumococcal infection, we studied the underlying molecular processes by which *S. pneumoniae* induces cell death. Specifically, we studied the pneumococcal toxin pneumolysin, which is present in almost all of the pathogenic pneumococcal strains^1^. While pneumolysin’s ability to kill cells and damage tissue is well established^12^,^13^,^14^,^15^,^16^, little is known about the underlying molecular processes that drive cytotoxicity. Recent studies point to DNA damage as a mechanism of pathogenicity during infection^20^,^21^. Here, we show that pneumolysin has a previously unidentified impact on genomic integrity and that pneumolysin-induced DNA damage is associated with cell cycle arrest and cytotoxicity.

To learn about the potential for pneumolysin to induce DNA damage, we monitored the formation of γH2AX repair foci that are known to form at sites of DSBs. We observed that pneumolysin is a potent inducer of DSBs. Further, pneumolysin-induced γH2AX foci are mediated by ATM and DNA-PK kinases, and they recruit 53BP1 and MDC1 to the sites of DSBs. At a clinically relevant concentration of pneumolysin (*i.e.* 100 ng/ml)^6^, the toxin was able to induce discrete repair foci at DSBs without any cell lysis, suggesting that pneumolysin’s genotoxicity can occur independently of its characteristic function as a cytolysin. Pneumolysin-induced DSBs caused cell cycle arrest, without significant apoptosis. In line with this observation, we found that inhibiting the NHEJ DNA repair pathway during pneumolysin exposure led to increased levels of toxicity and apoptosis. Further, we found that neutralizing the oligomerization domain of pneumolysin prevents pneumolysin-induced DNA damage and cell cycle arrest, indicating a potential role of pneumolysin oligomerization in activating host DNA damage response. Finally, we showed that pneumolysin is the key DNA damaging factor released during bacterial lysis. Overall, these results demonstrate the genotoxicity of pneumococcal pneumolysin and underscore the importance of DNA repair to counteract pneumolysin-mediated toxicity.

Pneumolysin is released from pneumococci when they are lysed^4^,^5^,^6^. Among the most common frontline antibiotics against *S. pneumoniae* are β-lactams^42^ which, while effective in killing the bacteria, lead to the release of pneumolysin, as has been demonstrated in both *in vitro* and *in vivo* models^6^,^43^. Hence, despite antibiotic-based treatment against bacterial pneumonia^44^, there are studies showing antibiotic therapy to be insufficient for treatment of patients in the intensive care unit^2^. In addition, significant concentrations of pneumolysin have been detected in cerebral spinal fluid of patients with pneumococcal diseases, sometimes up to 180 ng/ml^6^, which is significantly higher than the dose of pneumolysin that we showed here as genotoxic. Given pneumolysin’s acute pathogenicity, it is important to understand the molecular processes underlying the toxicity of pneumolysin.

CDC toxins are traditionally known for their role in cell lysis^45^. In the context of pneumolysin’s effect on nuclear DNA, one study demonstrated that pneumolysin specifically induces dephosphorylation of histone H3 at serine 10, through unidentified pathways, as a potential mechanism for host transcription regulation^46^. Here, we show that pneumolysin is able to induce H2AX phosphorylation at serine 139, a modification that is created along ~ 1–2 Mbp around a DSB^47^. There are potential mechanisms by which pneumolysin could be inducing DNA damage. While pneumolysin pore formation causes leakage of cytoplasmic protein and lysis via macropores (~26 nm), pneumolysin can also form ion channel-like micropores that can facilitate calcium influx^17^,^48^ and activate different signaling pathways^48^,^49^. Increased cytosolic calcium levels can stimulate the electron transport chain and membrane enzyme NADPH-oxidase to cause dysregulated production of intracellular reactive oxygen species (ROS)^50^, which can inflict direct DNA damage^51^. Indeed, ≤100 ng/ml of pneumolysin has been shown to elevate ROS production in neuronal cells^52^ and neutrophils^53^. Increased calcium levels can also induce DNA damage via activation of endonucleases that require calcium for their activity to cleave DNA strands^54^. Although changes in calcium flux could be a plausible reason for pneumolysin-induced DNA damage, further studies are warranted to elucidate the detailed mechanisms.

While intracellular ROS could mediate pneumolysin-induced DSBs, single strand breaks (SSBs) produced by ROS damage could also be a source of DSBs. SSBs encountered by replication forks can lead to replication fork breakdown, and if the SSBs are in close proximity, a DSB can be formed^39^, leading to γH2AX formation. We observed that at 12 hours there is a substantial population of γH2AX and EdU-positive cells. Importantly, at 48 hours, the dual positive cells are insignificant in numbers while the γH2AX positive cells persist. These results indicate that the observed increase in γH2AX is mainly induced by pneumolysin and not an intrinsic cell cycle event. Although our study did not address the direct role of replication stress on pneumolysin-induced H2AX phosphorylation, the observation that a sub-population of γH2AX-positive cells was also positive for EdU incorporation indicates that pneumolysin-induced DNA damage could arise during replication stress or fork breakdown.

PI3KKs play important roles in response to DNA damage^27^. We observed that increasing the concentration of inhibitors of ATM and DNA-PK (KU55933 and NU7441 respectively), increased the extent of ATP depletion and detachment of adherent epithelial cells during pneumolysin treatment. Importantly, disrupting the NHEJ repair process by inhibiting DNA-PK with NU7441, sensitized the pneumolysin-exposed epithelial cells to apoptosis. This observation that pneumolysin is able to induce apoptosis when DNA-PK mediated repair is inhibited, demonstrates that DSB repair pathway is essential against pneumolysin-induced genotoxicity. The toxic effect of NU7441 was also demonstrated by ATP reduction in epithelial cells that were not exposed to pneumolysin. ATM inhibition by KU55933^55^ resulted in decreased γH2AX formation by pneumolysin, which is consistent with a role for ATM in phosphorylating H2AX in response to pneumolysin-induced DSBs formation. Interestingly, DNA-PK inhibition by NU7441 was also able to significantly reduce pneumolysin-induced γH2AX foci formation. This could be partly attributed to the redundant ability of DNA-PK to generate γH2AX^32^. In addition, both NU7441 and KU55933 are designed as ATP-competitive inhibitors of DNA-PK and ATM, respectively. It is possible that using NU7441 throughout pneumolysin treatment, could have non-competitive and non-specific inhibition of related phosphatidylinositol-3’ kinases (including ATM and ATR) that phosphorylate H2AX. Taken together, these observations suggest that pneumolysin-induced DSBs activate ATM pathway and depend on DNA-PK-mediated NHEJ pathway for DNA repair in order to prevent apoptosis.

We observed that pneumolysin-induced DSBs in alveolar cells precede cell cycle arrest, which is possibly mediated by p21 pathway. During DNA damage, p53 regulates cell arrest and apoptosis^33^. During pneumolysin treatment, although p53 protein levels were not substantially elevated, p21 was significantly upregulated. Previous studies^34^,^35^ have shown that p53 is differentially activated in long and short pulses depending on the severity of DNA damage, to regulate either cell arrest and survival or cell death. p53 levels are greatly increased and sustained to execute apoptosis, however, lower levels of p53, possibly activated in short pulses, may contribute to cell cycle arrest^34^,^35^. Hence, one possible reason for absence of significant p53 induction in our observation could be that p53 is activated in short pulses to facilitate cell arrest until repair is completed^34^,^35^. Indeed, low levels of p53 have been demonstrated to induce p21 expression and cause cell cycle arrest^56^,^57^. Overall, the results shown here are consistent with a role for p53-p21 pathway in cell cycle arrest, leaving time for repair in order to prevent apoptosis.

With the prevalent use of bacteriolytic antibiotics in treatment of pneumococcal diseases, host cells are certain to be exposed to pneumolysin. Concentration of pneumolysin (<100 ng/ml) that does not lyse mammalian cells, is shown to induce production of inflammatory factors in immune cells^18^,^19^. Here, we show that pneumococcal lysis releases pneumolysin as a genotoxic factor, suggesting that the use of bacteriolytic antibiotics during pneumococcal diseases may exacerbate host DNA damage. Pneumolysin-induced DNA damage could aggravate the collateral damage done by ROS-producing inflammatory cells, culminating in increased genomic damage that could trigger cell death. Importantly, regeneration and repair of alveolar cells are critical to restore functional pulmonary architecture in the aftermath of pathogen-induced lung injury^58^. Pneumolysin-induced cell cycle arrest in alveolar cells could impair or delay tissue repair and regeneration processes during the recovery phase.

In summary, these studies reveal a previously unidentified ability of pneumococcal pneumolysin to induce DSBs, at pathologically relevant concentration. Interestingly, the genotoxicity of pneumolysin is dependent on its binding and oligomerization on the host cell membrane, and may not require membrane lysis. Importantly, we showed that pneumolysin-induced DSBs elicit DNA damage response that involves activation of NHEJ pathway to repair the DSBs and subsequent p21-mediated cell cycle arrest to allow time for DNA repair. Pneumolysin is closely related to other toxins of the CDC family such as listeriolysin, streptolysin and perfingolysin that are produced by various pathogenic bacteria^59^. Given that pneumolysin shares 40–70% sequence identity with these toxins^59^, this study encourages future investigation of these CDC toxins for potential genotoxicity during disease pathogenesis.

## Materials and Methods

### Cell culture

Human lung alveolar carcinoma (type II pneumocyte) A549 cell line was maintained in F12-K medium (Gibco) with 15% fetal bovine serum at 37 °C and 5% CO_2_. Cells were seeded at 2 × 10^5^ cells/ml for all experiments.

### Preparing bacterial protoplast lysate

Protoplasts were isolated as described previously^60^. Briefly, 2 ml of bacterial culture (serotype 3 Xen 10 A66.1, serotype 4 TIGR4 and serotype 19 F clinical isolate), grown in brain heart infusion broth (with serum) for 8 h, were centrifuged, washed with PBS and re-suspended in 100 μl of cell wall digestion buffer (10 mM Tris pH 7.5, 30% Sucrose, 1x protease inhibitor, 300 U/ml mutanolysin, 1 mg/ml lysozyme) for 3 h at 37 °C. The protoplasts were pelleted at 6000 rpm for 10 min at 4 °C, washed and resuspended in F-12 K media. Protoplasts were lysed with 100 μl volume of 0.1 mm silica beads (Biospec) in Tissuelyser II (Qiagen) at 30 beats/sec for 10 min. The lysate was centrifuged and the supernatant was filter sterilized, made up to 1 ml with F-12 K medium and incubated with A549 cells. The supernatant was also plated out to confirm the absence of bacterial colonies.

### Treatment of cells

Chemical inhibitors KU55933 and NU7441 (Selleckchem) were dissolved in DMSO and stored as 2 mM stock. Each inhibitor (10 or 20 μM), dissolved in F12-K media, was incubated with A549 cells for up to 6 h. To detect DNA replication, 10 μM EdU (Invitrogen) was incubated in F12-K medium for 30 min at 37 °C. ATP level in cell culture was determined in 96-well plate by using Cell Titre-Glo luminescent assay (Promega).

### Recombinant pneumolysin

pET101 vector containing pneumolysin sequence with C-terminal His-tag was a generous gift from Prof. Larry S. McDaniel (University of Mississippi Medical Center). The plasmid was transformed into competent E. coli One shot BL21 (DE3) (Invitrogen) for recombinant protein expression by 1 mM IPTG induction. The bacterial pellets were sonicated in buffer (100 mM Potassium Phosphate buffer pH 8, 300 mM NaCl, Protease inhibitor) at 6 sec pulse on and off cycle (25–30% amplitude, Sonics Vibracell) for 30 min, keeping the suspension in ice. The sonicated suspension was centrifuged at high speed and supernatant was subjected to binding with equilibrated Ni-NTA slurry (Qiagen) and 25 mM imidazole at 4 °C for 4 h. The slurry was then loaded into a column, and washed with buffer (20 ml for 40 ml lysate). The recombinant pneumolysin bound to the column was then eluted using increasing concentrations of imidazole (75, 100, 150, 200 mM). Protein purity of each elution was checked using SDS-PAGE. The elutions containing pure protein (single band in SDS-PAGE) were pooled and dialyzed overnight at 4 °C in 10 kDa membrane against 50 mM Tris-Cl pH 8 and 20% glycerol. Recombinant protein was concentrated using an Amicon ultra filter (3 kDa cutoff). The final protein concentration was determined by Biorad protein assay, diluted with buffer (50 mM Tris-Cl pH 8, 150 mM NaCl, 20% glycerol), aliquoted and stored at −20 °C.

### Pneumolysin Neutralization assay

The neutralizing effect of anti-pneumolysin monoclonal antibody was tested against 25 ng/ml pneumolysin. Antibody was diluted to 100 μg/ml, and then serially diluted at 1:2 ratio. Each antibody dilution (100 μl) was added to recombinant pneumolysin for 5 min. RBCs was added to the mixture, and incubated at 37 °C for 20 min. The cells were centrifuged and supernatant was measured at OD_541_ nm. The percentage hemolysis was calculated as the percentage of total lysis by RBC lysis buffer (Gibco).

### Immunofluorescence

After incubation with pneumolysin, cells were permeabilized with 0.2% Triton X-100 in PBS for 10 min, and blocked with 3% bovine serum albumin (BSA) in PBS for 40 min. Primary antibodies against γH2AX (Ser-139) (Millipore #05–636), 53BP1 (Santa Cruz #sc-22760), MDC1 (Abcam #1169), p53BP1 (S1778, Cell signaling #2675) were used at 1:100 dilutions in PBS, and incubated for 1 h at room temperature with cover slips. For TUNEL staining, the labeling enzyme (Roche #1 1684795 910) was incubated similarly for 1 h at 37 °C. Secondary antibodies (Invitrogen) labeled with either Alexa Fluor 488 or 564 or 647 were used. For EdU detection, Alexa Fluor azide was used in Click-iT reaction buffer (Invitrogen). Slides were mounted with SlowFade (Invitrogen) and stored at 4 °C until imaging. All stained slides were examined under confocal microscope, and 15 images (at 5 × 3 sites) were taken for each well under 40X magnification.

### DNA damage quantification

Cell imaging and enumeration were performed using Zeiss Zen microscope in the dark room. Cells within 15 images per condition were counted. Cells were observed as those with (a) >5 distinct foci of γH2AX or 53BP1 (b) nuclei with colocalized foci of γH2AX and 53BP1, or colocalized γH2AX and EdU. Cells that showed colocalization of FITC and DAPI were counted as TUNEL positive cells. Nuclei were selected by DAPI channel alone, and subsequently analyzed under fluorescent channels.

### Cell arrest analysis

Trypsinized cells were washed and resuspended in 1% paraformaldehyde and placed on ice for 1 h. After washing with PBS, cells were resuspended in 200 μl PBS and added with 1 ml of 70% ethanol (dropwise) and kept overnight at 4 °C. Cells were washed and resuspended in PBS containing 60 μg/ml propidium iodide and 100 μg/ml RNAse for 30 min at 37 °C. After wash, the cells were analyzed by flow cytometer.

### Western analysis

Treated cells were washed with PBS, and incubated for 10 min with 200 μl of 1 x lysis buffer (50 mM Tris-HCl pH 6.8, 25 mM dithiothrietol, 2% SDS, 10% glycerol). The lysate was centrifuged for 10 min at 4 °C and the supernatant was denatured at 100 °C for 10 min and stored at −20 °C. Protein concentration was quantified using the BioRad DC protein assay kit, and lysates were electrophoresed in 10% SDS-PAGE. Analysis was done using anti-p53, anti-p21 and β-actin (Santa Cruz) antibodies and with secondary antibody conjugated with HRP (Dako), and later developed by adding Amersham ECL prime reagent (GE Life Science). Blots were exposed on Kodak film and band intensities were quantified using Thermo Scientific myImageAnalysis v1.1.

## Additional Information

**How to cite this article**: Rai, P. *et al.* Pneumococcal Pneumolysin Induces DNA Damage and Cell Cycle Arrest. *Sci. Rep.* **6**, 22972; doi: 10.1038/srep22972 (2016).

## Supplementary Material

Supplementary Information
