# Supplementary material for: Pneumococcal Pneumolysin Induces DNA Damage and Cell Cycle Arrest
Source: Sci Rep. 2016 Mar 30;6:22972. doi: 10.1038/srep22972 (PMC4812240; doi:10.1038/srep22972)
Supplement: Supplementary Information [file srep22972-s1.pdf]

Pneumococcal Pneumolysin Induces DNA Damage and Cell Cycle Arrest

Prashant Rai [1,2], Fang He [3], Jimmy Kwang [2,3], Bevin P. Engelward [1,4,¶] and Vincent T.K. Chow[1,2,¶,*]

[1] Infectious Diseases Group, Singapore-MIT Alliance for Research & Technology, Singapore 138602

[2] Department of Microbiology and Immunology, Yong Loo Lin School of Medicine, National University of Singapore, Singapore 117545

[3] Animal Health Biotechnology, Temasek Life Sciences Laboratory, National University of Singapore, Singapore 117604

[4] Department of Biological Engineering, Massachusetts Institute of Technology, Cambridge, MA 02139

[¶] Both authors contributed equally.

[*] Corresponding author.

Addresses:

Vincent T.K. Chow, Department of Microbiology and Immunology, Yong Loo Lin School of Medicine, 5 Science Drive 2, Kent Ridge, National University of Singapore, Singapore 117545.

E-mail: micctk@nus.edu.sg

Bevin P. Engelward, Department of Biological Engineering, Massachusetts Institute of Technology, Cambridge, MA 02139, USA.

E-mail: bevin@mit.edu

A

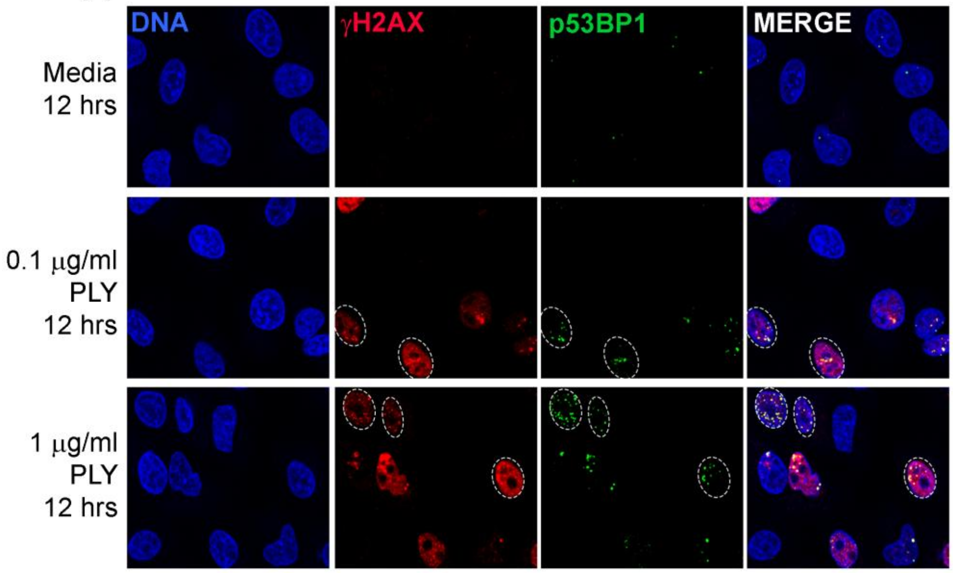

Supplementary Figure 1 Recombinant pneumolysin was incubated with alveolar epithelial cells at concentrations of 0.1 μg/ml and 1 μg/ml for 12 h, and analyzed for MDC1 and phospho (Ser 1775)-53BP1 (p53BP1). Representative images show nuclei (blue), γH2AX (red) with p53BP1 (green) and colocalization foci (yellow). Media indicates negative control without any pneumolysin. Images are from three independent experiments.

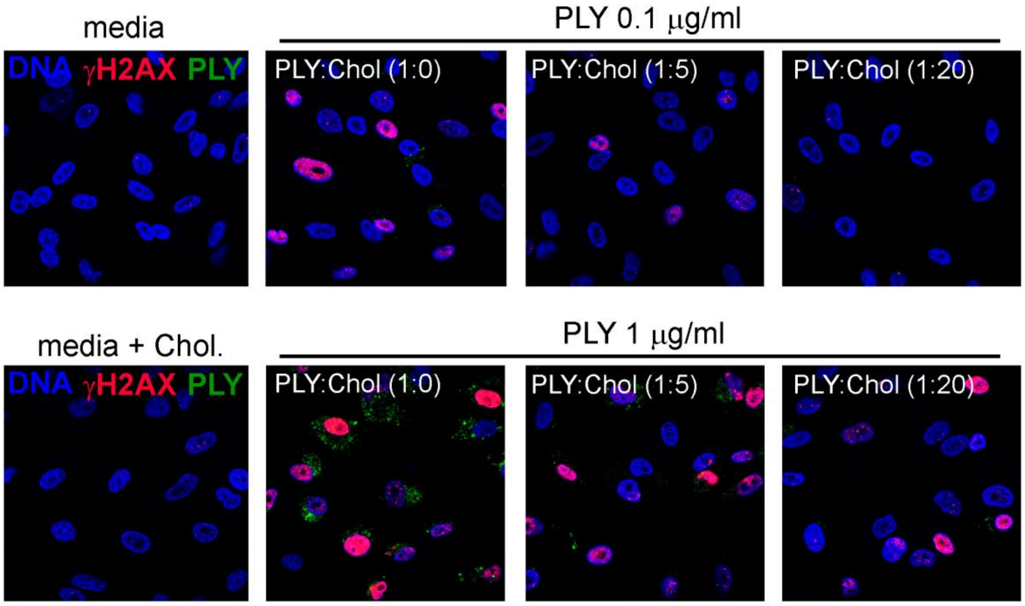

Supplementary Figure 2 Pretreatment of pneumolysin with cholesterol reduces genotoxicity. Pneumolysin was incubated with cholesterol at mass ratio (PLY:Chol) of 1:5 and 1:20 for 20 min at 37°C. Control without cholesterol treatment is indicated as Ply:Chol (1:0). Treated pneumolysin was then diluted and incubated with alveolar epithelial cells for 12 hrs and analyzed for γH2AX. Representative images showing nuclei (blue), γH2AX (red) and pneumolysin (green). Images are representative from three independent experiments..
